# Loading of ”cocktail siRNAs” into extracellular vesicles via TAT-DRBD peptide for the treatment of castration-resistant prostate cancer

**DOI:** 10.1080/15384047.2021.2024040

**Published:** 2022-02-16

**Authors:** Yanjun Diao, Gangqiang Wang, Bingbing Zhu, Zhuo Li, Shan Wang, Lijuan Yu, Rui Li, Weixiao Fan, Yue Zhang, Lei Zhou, Liu Yang, Xiaoke Hao, Jiayun Liu

**Affiliations:** a Department of Clinical Laboratory Medicine, Xijing Hospital, Fourth Military Medical University, Xi’an, Shaanxi, China; b Department of Laboratory Medicine, The First Affiliated Hospital of Xi’an Medical University, Xi’an, Shaanxi, China; c Department of Clinical Laboratory Medicine, The Fourth Hospital of Xi’an, Xi’an, Shaanxi, China

**Keywords:** Prostate cancer, extracellular vesicles, siRNA delivery, hormonal resistance, multi-targets therapy

## Abstract

Extracellular vesicles (EVs) are cell-derived, membranous nanoparticles that mediate intercellular communication by transferring biomolecules between cells. As natural vehicles, EVs may exhibit higher delivery efficiency, lower immunogenicity, and better compatibility than existing RNA carriers. A major limitation of their therapeutic use is the shortage of efficient, robust, and scalable methods to load siRNA of interest. Here, we report a novel strategy using polycationic membrane-penetrating peptide TAT to encapsulate siRNAs into EVs. Three TAT peptides were co-expressed with DRBD as 3TD fusion protein. The sequence-independent binding of DRBD facilitates multiplex genes targeting of mixed siRNAs. Functional assays for siRNA-mediated gene silencing of CRPC were performed after engineered EVs treatment. EVs were isolated using differential centrifugation from WPMY-1 cell culture medium. The increase of merged yellow fluorescence in the engineered EVs showed by TIRFM and the decrease in zeta potential absolute values certified the co-localization of siRNA with EVs, which indicated that siRNA had been successfully delivered into WPMY-1 EVs. qRT-PCR analysis revealed that the mRNA level of FLOH1, NKX3, and DHRS7 was dramatically decreased when cells were treated with engineered EVs loaded with siRNAs mixtures relative to the level of untreated cells. Western and flow cytometry results indicate that delivery of siRNA mixtures by engineered EVs can effectively downregulate AR expression and induce LNCaP-AI cell apoptosis. The uptake efficiency of the EVs and the significantly downregulated expression of three genes suggested the potential of TAT as efficient siRNA carriers by keeping the function of the cargoes.

## Introduction

It has also been shown that EVs naturally carry nucleic acids, such as DNA and RNA, to targeted cells, inducing genetic modifications in biological and pathogenic processes. Based on these features of EVs, studies have been conducted using EVs as therapeutic small interference RNA (siRNA) delivery systems to alter gene expression in certain diseases and improve genetic therapy.^1,2^ Compared with other siRNA delivery strategies such as viruses, liposomes, and polycationic polymers, EVs are non-immunogenic in nature due to their similar composition as body cells. Besides, EVs can act as therapeutic vehicles that help protect against quick degradation of siRNA in the systemic circulation, which offers significant advantages in the exploitation of therapeutic siRNA drugs. However, determining the utility of EVs to deliver exogenous siRNA still requires much more investigation and understanding. Considering the low output of EVs, it is urgent to find a safe, efficient, and non-damaging siRNA loading method using it as the delivery vehicle. At present, chemical transfection and electroporation are currently the two main loading methods. The results showed that chemical transfection was unsuitable and the data were inconclusive.^3^ Although electroporation is effective, it inevitably causes damage to the membrane of the exosome. The purpose of the study was mainly to find a suitable method to deliver siRNA to EVs and test the usefulness of EVs as therapeutic vehicles in cancer therapy.

**Figure 1.**
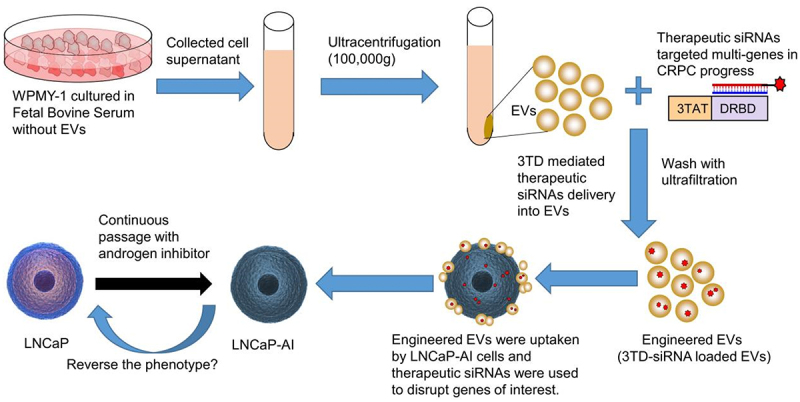
Schematic of strategy to load siRNAs into EVs and predicted treatment effect of engineered EVs on CRPC cells.

Advanced prostate cancer (PCa), which is initially sensitive to androgen deprivation therapy, usually progresses into castration-resistant prostate cancer (CRPC) ultimately. However, apart from the well-acknowledged androgen receptor (AR) signaling pathway, this progress involves multiple genes and multiple mechanisms. Many studies have demonstrated the significant benefit of multi-targeted siRNA inhibitors compared with single gene-targeted inhibitory drugs.^4^ Cocktail siRNAs targeting multiple genes may enhance the anticancer effect as a therapeutic approach if all the distinct targets are CRPC-related genes.

Here, we would like to use the widely recognized cell-penetrating peptide TAT, a peptide domain of HIV1 trans-activator for transcription, to mediate siRNA into EVs efficiently through micropinocytosis. The combination of TAT and siRNA was achieved by a double-strand RNA binding domain (DRBD). The binding of siRNA mediated by DRBD was RNA sequence-independence, which provided unique advantages for therapeutic siRNA against multiple castration-resistant genes. Consequently, the objective of this study was to construct an engineered EV by exploring TAT-DRBD recombinant protein to mediate cocktail siRNA loading and assess its treatment effect on CRPC cells (Figure 1).

## Methods

### Materials

Unless otherwise noted, all primers and restriction enzymes were purchased from Takara Biotechnology (catalog number Xho I 1635, Sma I 1629). All primary antibodies were purchased from Abcam: Anti-HIV TAT (N3), ab63957; EVs Anti-CD63[EPR5702], ab134045; Anti-ALIX [3A9], ab117600; Anti-Androgen Receptor (AR-V7 specific) [EPR15656], ab198394. The secondary antibody was purchased from Odyssey (Goat anti-Mouse antibody, IRDye 680). Lipofectamine 3000 Transfection Reagent was purchased from Life Technology (L3000008). Plasmid pET-44bC vector was purchased from Novagen (71123–3). T4 DNA ligase was purchased from New England BioLabs (M0202S). His GraviTrap Kit for protein purification was purchased from GE Healthcare (11–0036-90 AA). TRIzol was purchased from Life Technology (15596026). Reverse transcription kit and real-time quantitative PCR kit were purchased from Takara Biotechnology (RR036A and RR820A). Cell Counting Kit-8 (CCK8) was purchased from Dojindo (CK04). Negative control siRNA (siRNA-NC), Cy5-labeled control siRNA (Cy5-siRNA), FAM-labeled control siRNA (FAM-siRNA), therapeutic siRNAs targeting FLOH1, NKX3, DHRS7 genes, and the negative control siRNA were synthesized by RiboBio Co., Ltd. All other reagents and chemicals were of analytical grade and used without further purification.

### Cell culture

Dulbecco’s modified Eagle’s medium (DMEM), phenol red-free DMEM, fetal bovine serum (FBS), charcoal-stripped FBS (CS-FBS), penicillin, streptomycin, 0.25% trypsin and phosphate-buffered saline (PBS) were purchased from Gibco (Gaithersburg, MD, USA). WPMY-1 (Human Normal Prostate matrix Immortalized Cells) and LNCaP cells (androgen-dependent human prostate cancer cell line) were purchased from American Type Culture Collection (ATCC®, CRL-2854™, CRL-1740). The androgen-independent human prostate cancer cell-line LNCaP-AI was a gift from the Department of Laboratory Medicine, The Second Affiliated Hospital of Zhejiang University.^5,6^ WPMY-1 and LNCaP cells were grown in RPMI 1640 supplemented with 10% FBS (Gibco, 26140079). To produce EVs, the culture medium of WPMY-1 cells was replaced with RPMI 1640 supplemented with 10% Exosome Depleted FBS (VivaCell Bioscience, C38010050) when grown to 30–40% density. LNCaP-AI cells were maintained in phenol red-free DMEM/F-12 (Gibco, 12400024) with 10% CS-FBS (Gibco, 12676–029). All cells were cultured at 37°C and 5% CO2 supplemented with 1% penicillin–streptomycin (Gibco, 15140122).

### Preparation of each fraction

#### Preparation of EVs

WPMY-1 cells were cultured in exosome-free RPMI 1640 medium for 48 h. The supernatant was collected and then centrifuged at 300 × g for 10 min to remove cells, 2,000 × g for 10 min to remove dead cells, and 10,000 × g for 30 min at 4°C to deplete residual debris. Afterward, the EVs were pelleted by ultracentrifugation at 100,000 × g for 70 min at 4°C and then washed with PBS (depleted with original supernatant and changed with PBS). The EVs were finally pelleted by the second ultracentrifugation at 100,000 × g for 70 min at 4°C and were resuspended in PBS. The particle size, distribution, and concentration of EVs were determined by NTA, and the EVs solution was aliquoted and stored at −70°C.

#### Expression and purification of 3TD fusion protein

The DNA fragments of TAT-TAT-TAT-DRBD (3TD in abbreviation) were synthesized by Sangon Biotech and inserted between Sma I and Xho I of pET-44b, downstream of a soluble Nus Tag and upstream of a His Tag. Plasmid pET44b-3TD was transformed into E. coli strain Rosetta-gami, and the bacteria were cultured 6 hours in Luria Broth at 25°C with 0.1 mM IPTG. The E. coli pellet was disrupted by sonication and the 3TD fusion protein was purified from the supernatant using Ni-NTA affinity chromatography. The purified product was analyzed by SDS-PAGE and Western blot using LI-COR Odyssey infrared fluorescence imaging system. The eluted fraction was dialyzed to remove imidazole with ultrafiltration. The freeze-dried 3TD protein was redissolved and the concentration was adjusted to 1 μg/μL with PBS and stored at −70°C for further usage.

### Construction of engineered EVs (3TD-siRNA loaded EVs)

#### Interaction between 3TD fusion protein and siRNAs

DRBD binds to a dsRNA backbone on 90 surface quadrants of the dsRNA helix, resulting in 4 DRBDs encompassing a single-molecule siRNA.^7^ Theoretically, these will form a complex at a ratio of 4:1 for 3TD protein and siRNA. siRNA with random sequence was labeled with Cy7 when synthesized by RiboBio Co. Ltd. and stored at 20 mM. To verify the combination of 3TD and siRNA, we incubated 10 μL siRNA (diluted to 1/32 μM) with 3TD fusion protein in PBS with 10% glycerol on ice for 2 h. 3TD fusion protein (20 μg) was 2-folds diluted up to 1/128. Then, 5 × Electrophoretic Mobility Shift Assay (EMSA)/Gel-Shift binding buffer (Beyotime Biotechnology, GS002) was added to the reaction before siRNA were shifted in 2% agarose gel. Bull Serum Albumin (BSA) without DRBD was used as a negative control. The result was analyzed using LI-COR Odyssey infrared fluorescence imaging system.^8^

#### Verification of EVs siRNA-loading effect of 3TD

To determine whether the 3TD protein could efficiently deliver siRNA into EVs, we evaluated the loading effect by the co-localization of EVs and Cy5.5-siRNA using a total internal reflection fluorescence microscope (TIRFM). Before use, the concentration of re-thawing EVs aliquots was adjusted to 1E+10 particles/mL with PBS. Briefly, the control group with 200 μL EVs mixed with 200 μL free siRNA-Cy5.5 directly and the engineered group with siRNA-Cy5.5 loaded into EVs by 3TD were mixed gently with 500 μL Diluent C solution separately, and then incubated on ice for 5 min. Meanwhile, 1 μL PKH dye was diluted with 500 μL Diluent C solution in another tube. The two solutions were mixed and incubated on ice for 5 min. 1% BSA solution was used to stop the staining. After that, free siRNA-Cy5.5 attached to the surface of EVs was removed by incubating the samples with RNase A for 1 h. Then, the siRNA-Cy5.5 loaded EVs were pelleted by ultracentrifugation and resuspended in 20 μL PBS. They were then visualized with tethered cationic lipoplex nanoparticles biochip^9,10^ and a Nikon TIRFM. Moreover, the co-localization percentage was calculated by equation % = Cy5.5 containing EVs/total EVs labeled by PKH dye. To further enhance the specificity of EVs characterization, CD63 antibody with green fluorescence was used to mark engineered EVs in the RNase digestion experiment, and the magnified images of EVs before and after RNase A digestion were collected to show the localization of siRNA.

In addition, the negative charges of EVs will be affected by loaded siRNA or TAT. The zeta potential of EVs with different loading strategies was obtained by dynamic light scattering goniometry (DLS). Samples were diluted in 20 mM HEPES buffer. The wavelength of the laser was set at 632.8 nm and the detection angle at 90°at 20°C. Three independent measurements, each composed of 10 runs, were carried out, and the zeta potential values were presented as mean ± standard deviation (n = 3).

#### Cellular uptake and intracellular location of engineered EVs in LNCaP-AI cells

Confocal Laser Scanning Microscopy (CLSM) was used to observe the distribution of siRNA-FAM in LNCaP-AI cells. A control group (LNCaP-AI cells + the mixture of EVs&free siRNA-FAM) was set up to compare with the experimental group (LNCaP-AI cells + engineered EVs). After a 6 h incubation, the LNCaP-AI cells were washed twice with cold PBS and fixed with 4% formaldehyde in PBS for 30 min. Nucleic acids of LNCaP-AI cells were stained with 5 μg/ml of DAPI for 5 min, and F-actin was stained with 100 nM rhodamine-conjugated phalloidin for 30 min at room temperature. Excess dyes were washed three times with PBS prior to microscopy. Images were captured on an Olympus FV10i confocal microscope at 240-folds magnification.

To further confirm the intracellular siRNA-transfection efficiency of engineered EVs, flow cytometry was used to quantitatively analyze cellular fluorescence. LNCaP-AI cells were seeded at a density of 5 × 105 cells/well in a 6-well plate 24 h prior to the experiment. Cells were treated with either free siRNA-FAM, EVs, and siRNA-FAM mixture or engineered EVs (3TD-siRNA-FAM loaded EVs) for 48 h. After trypsinization, cells were washed three times with cold PBS (pH = 7.4) and fixed in 70% ethanol. After fixation, cells were incubated with 100 U/ml RNase A at 37°C for 20 min. >5,000 cells from each group were collected and analyzed using flow cytometry (FL1-H channel) to test the mean fluorescence intensity (MFI).

#### Characterization of engineered EVs

Subsequent to the construction, the main performance parameters of engineered EVs were detected as follows. Transmission electron microscopy (TEM) was performed to observe the morphology of engineered EVs. Further identification of engineered EVs was made by Western blot, examining the expressions of TAT and EVs maker proteins CD63 and ALIX. Particle size and concentration were determined using nanoparticle tracking analysis (NTA), and the zeta potential was calculated using dynamic light scattering (DLS).

### Evaluation of treatment effects

#### Selection of targeted genes in CRPC progress

Genes of interest are critical in evaluating the effectiveness of siRNA inhibitory therapy. Considering the complexity of mechanisms and genes in CRPC progress, a more powerful approach is to search for the unique genes that were shared in all the three reported CRPC genes models.^11–13^ Unfortunately, no genes with good evidence have been found. In addition, TCGA database was used to investigate further the expression and clinical significance of all the candidate genes in the three models. Essential driver genes with high prostate tissue specificity and elevated expression in high Gleason Score (GS) PCa were selected.

#### Validation of gene silencing effect of engineered EVs

Prior to incubating EVs with LNCaP-AI cells, an equal volume of each siRNA (2 μg/μL) was mixed, and the mixture was delivered by 3TD to construct the engineered EVs comparing the “therapeutic engineered EVs” group with the “no therapeutic control EVs” group. qRT-PCR was used to validate the RNA interference effect using iScript One-Step RT-PCR Kit with SYBR Green (Takara) 48 h after transfection. The results of three independent experiments were normalized relative to that of β-actin to correct for differences in template input applying 2^−ΔΔ^CT algorithm. Furthermore, the expression of AR, which is generally considered to have an early expression response to androgen treatment, was analyzed by Western blot.

To further determine the apoptosis-inducing effect of gene silencing, LNCaP-AI cells were transfected with engineered EVs at 70% confluency. 72 h later Annexin V-FITC/PI method (BD, 556547) using flow cytometry was adopted to measure cell apoptosis. A control EVs group loading with siRNA-NC was used to test if the apoptosis was included by successfully targeting the genes of interest.

### Statistical analysis

Data were presented in the form of mean ± SE. Statistical analysis was performed using a two-tailed Student’s t-test, and differences with a P value less than 0.05 were designated statistically significant.

## Results

### Preparation of each fraction

#### Production of EVs from WPMY-1 supernatant

**Figure 2.**
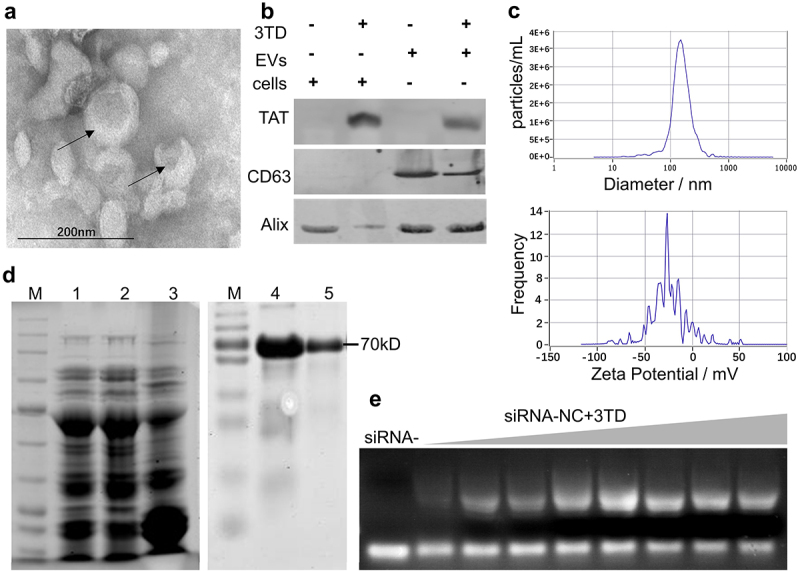
Preparation of EVs and construction of the 3TD-siRNA complex. (a) TEM image of WPMY-1 EVs collected by ultrafiltration. (b) Protein isolated from the engineered EVs or the EV producing WPMY-1 cells were detected by Western blotting using antibodies against the various proteins shown. Cells and EVs were treated with 3TD protein respectively. (c) Particle size and Zeta potential distribution of WPMY-1 EVs as determined by NTA and DLS. (d) SDS–PAGE and Western blotting analysis of purified 3TD protein. Lane M: standard molecular weight markers of protein; Lane 1: whole bacteria of E. coli Rosetta-gami transformed with pET-44b-3TD after boiled lysis; Lane 2 and 3: supernatant and precipitate of bacteria above after ultrasonic lysis. Lane 4: 3TD protein in ultrasound supernatant corresponding to Lane 2 with anti-TAT antibody; Lane 5: purified 3TD protein using Ni-NTA affinity chromatography column with anti-TAT antibody. (e) EMSA analysis of the interaction between 3TD fusion protein and siRNAs. 10 μL siRNA (1/32 μM) pre-incubated with serial 2-fold diluted 3TD protein. Untreated siRNA was used as mock control and Bovine Serum Albumin (BSA) as a negative control.

To obtain a pool of safe cell-derived EVs for gene therapy, we cultured WPMY-1, a non-tumorigenic human normal prostate matrix immortalized cells, in large quantities. Then, EVs from its supernatant were purified by ultracentrifugation. Representative TEM and Western blot image of EVs preparations is shown in Figure 2a,b. The particles had a mean diameter of 144.9 ± 15.6 nm and an average zeta potential of −22.80 ± 0.89 mV (Figure 2c).

#### Expression of 3TD fusion protein

A protein was purified using a Ni-NTA affinity chromatography column from the supernatant containing soluble lysate of the transformed E. coli. As expected, 3TD fusion protein was approximately 70 KDa as shown on SDS-PAGE and was identified by anti-TAT antibody (Figure 2d). Moreover, the siRNA binding effect of 3TD was tested by EMSA assay. Compared with free siRNA, agarose gel electrophoresis showed that 3TD blocked siRNA migration and the blocking effect was protein concentration-dependent (Figure 2e).

### Construction of engineered EVs

#### Verification of EVs siRNA-loading effect of 3TD

**Figure 3.**
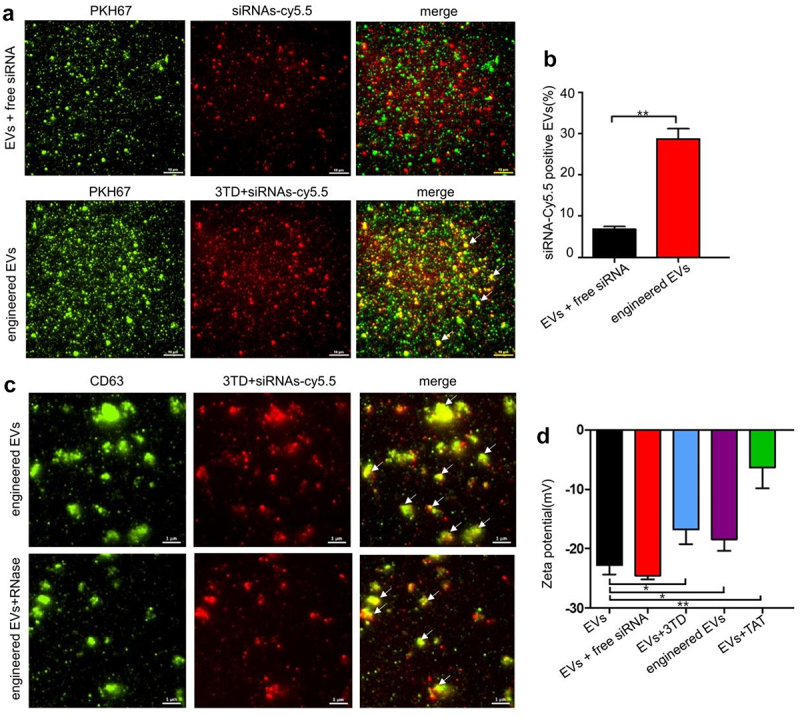
Verification of EVs siRNA-loading effect of 3TD. (a) Co-localization analysis of EVs and siRNA-Cy5.5 by TIRFM. Scalebar = 10 μm. (b) Percentage of EVs with siRNA-Cy5.5 fluorescence. Engineered EVs vs EVs+siRNA mixture, ** P < .01. (c) Location of siRNA-Cy5.5 in engineered EVs by TIRFM. Engineered EVs were showed before and after RNase digestion. Scalebar = 1 μm. (d) Zeta potential (mV) of engineered EVs before and after siRNA loading detected by DLS. N = 3, mean ± SEM. EVs: −22.80 ± 0.89, EVs+siRNA: −24.54 ± 0.36, EVs+3TD: −16.78 ± 1.45, engineered EVs: −18.41 ± 1.13, EVs+TAT: −6.347 ± 2.01.

Efficient loading of siRNA is key to turn EVs into therapeutic siRNA delivery vehicles. 3TD was adopted to permeabilize the EVs for siRNA loading. The siRNA was labeled with red fluorescence Cy5.5, and the EVs were stained with PKH67. The yellow fluorescence (white arrow in Figure 3a) in the engineered EVs captured by TIRFM showed siRNA co-localization with EVs, which indicated that siRNA has been successfully delivered into WPMY-1 EVs. In comparison, the co-localization percentage was significantly higher than that of the free siRNA control group (Figure 3b). The red fluorescence intensity (siRNA-cy5.5) of engineered EVs in the RNase digestion experiment was not significantly weakened after EVs were treated with RNase, which proved that siRNA has been successfully delivered to the inside of vesicles by 3TD, instead of attaching to the surface of EVs (Figure 3c). Furthermore, the negative surface charge of EVs would be affected after the loading of arginine-rich and cationic TAT peptides. The decrease of zeta potential absolute values in the engineered EVs group indicated the successful introduction of 3TD-siRNA into EVs (Figure 3d). In addition, the presence of TAT in engineered EVs validated by Western blot reconfirmed the above conclusion (Figure 2b).

### Cellular uptake of engineered EVs in LNCaP-AI cells

**Figure 4.**
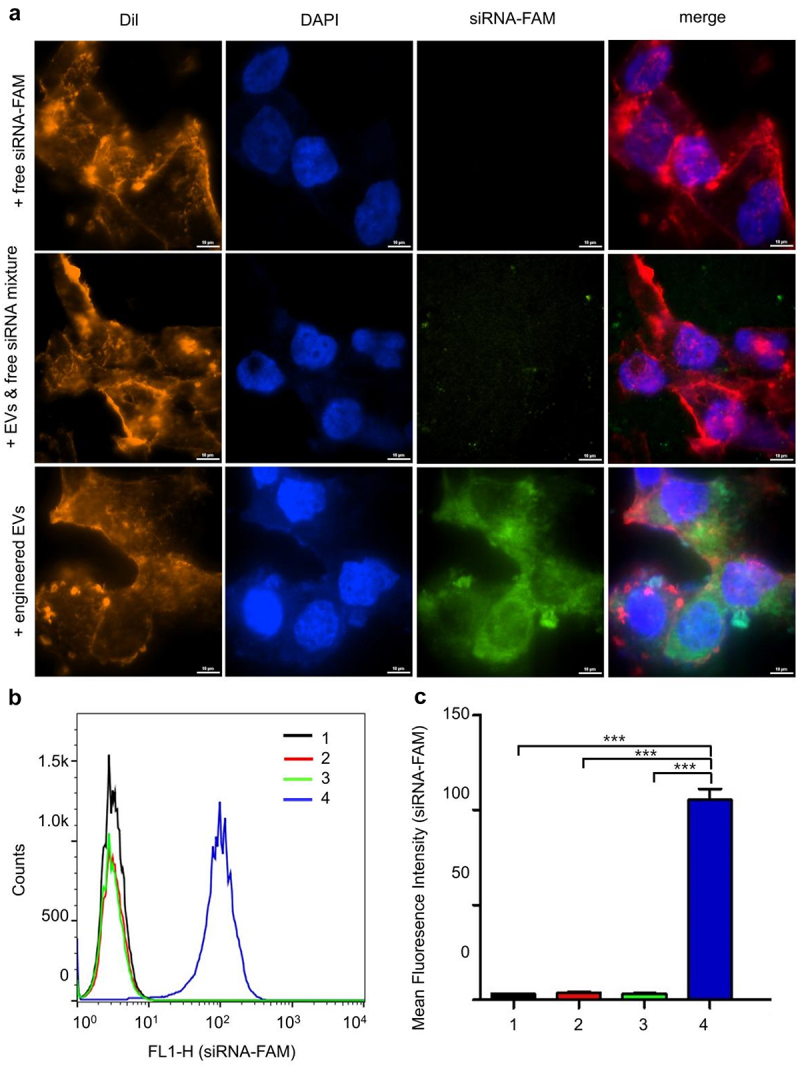
Cellular uptake and intracellular location of engineered EVs in LNCaP-AI cells. (a) Intracellular localization of engineered EVs by confocal microscopy. Red: Dil staining; Blue: DAPI staining; Green: FAM-siRNA. Scale bar: 10 μm (b) LNCaP-AI cells were treated with various groups at 37°C for 4 h, and FCS recorded cellular FAM fluorescence. X-axis: cellular fluorescence intensity; Y-axis: cell counts. (c) FAM Mean fluorescence intensity (MFI) was measured for different treatment groups. 1:+ PBS only; 2: +free siRNA-FAM; 3: EVs+siRNA-FAM mixture; 4: engineered EVs: 3TD-siRNA-FAM loaded EVs, n = 3. ***,P < .001.

To investigate the uptake of siRNA-loaded EVs by LNCaP-AI cells, confocal microscopy was performed 4 h after transfection of cells with siRNA-FAM loaded EVs. Results from the engineered EVs group showed extensive internalization and accumulation of green fluorescence in the cytoplasm of the recipient cells. In contrast, the free siRNA group and EVs & siRNA mixture group showed almost no fluorescence in the cytoplasm (Figure 4a). To confirm the delivery efficiency, we further verified the result by flow cytometry. The LNCaP-AI cells exhibited markedly increased fluorescence intensity in the engineered EVs group compared to the other three groups (Figure 4b,c). These data suggest that the incorporation of siRNA into EVs significantly improves the cellular uptake of therapeutic siRNA.

### Evaluation of treatment effect of engineered EVs on LNCaP-AI cells

#### Selection of multiple-targeted genes in CRPC progress

**Figure 5.**
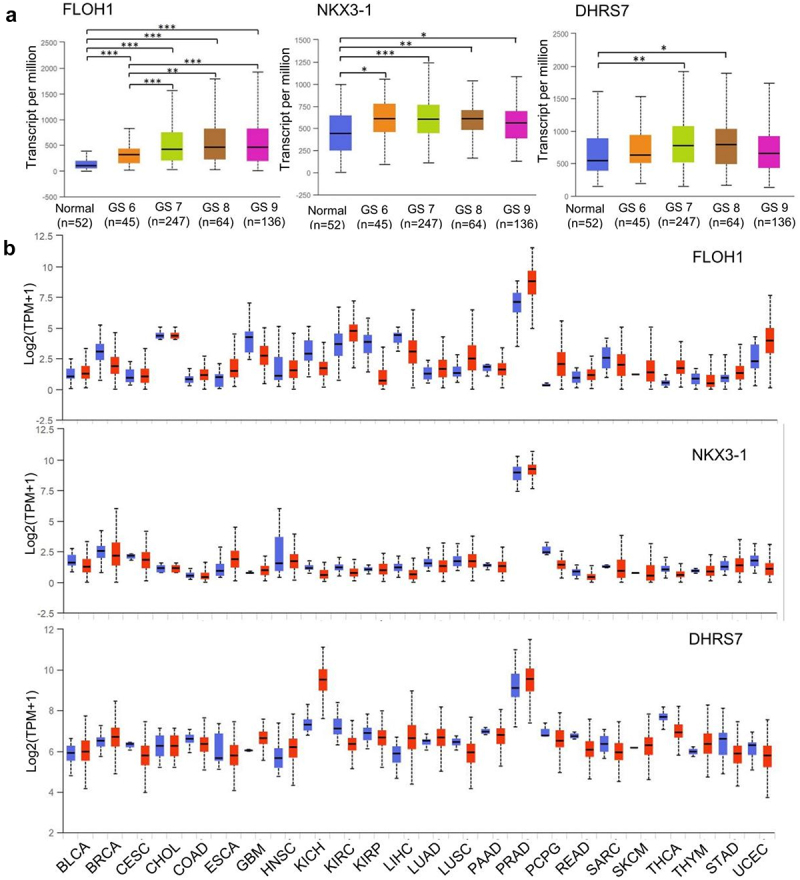
Expression of multiple-target genes in TCGA. (a)Expression of FLOH1, NKX3 and DHRS7 genes in PRAD based on patient’s GS. *,P < .05; **,P < .01; ***,P < .001. (b) Expression of FLOH1, NKX3 and DHRS7 genes across TCGA cancers. Blue bar: normal samples; Red bar: tumor samples. BLCA: Bladder Urothelial Carcinoma; BRCA: Breast invasive carcinoma; CESC: Cervical squamous cell carcinoma and endocervical adenocarcinoma; CHOL: Cholangiocarcinoma; COAD: Colon adenocarcinoma; ESCA: Esophageal carcinoma; GBM: Glioblastoma multiforme; HNSC: Head and Neck squamous cell carcinoma; KICH: Kidney Chromophobe; KIRC: Kidney renal clear cell carcinoma; KIRP: Kidney renal papillary cell carcinoma; LIHC: Liver hepatocellular carcinoma; LUAD: Lung adenocarcinoma; LUSC: Lung squamous cell carcinoma; PAAD: Pancreatic adenocarcinoma; PRAD: Prostate adenocarcinoma; PCPG: Pheochromocytoma and Paraganglioma; READ: Rectum adenocarcinoma; SARC: Sarcoma; SKCM: Skin Cutaneous Melanoma; THCA: Thyroid carcinoma; THYM: Thymoma; STAD: Stomach adenocarcinoma; UCEC: Uterine Carcinosarcoma.

Three target genes FLOH1, NKX3, and DHRS7 were screened from the TCGA database (544 samples). FLOH1 showed a significantly elevated expression in PCa with high GS compared with low GS, which has been proved to be a potential target to repress the abnormal proliferation in CRPC (Figure 5a).^14,15^ NKX3 is a unique androgen-regulated transcription factor, which functions as a negative regulator of epithelial cell growth in prostate tissue and coordinates AR to facilitate CRPC.^16–19^ DHRS7 encodes short-chain dehydrogenases/reductases, which are involved in steroid hormone synthesis and metabolism.^20^ All the three genes were specifically highly expressed in PCa across TCGA cancers (Figure 5b). The sequence of therapeutic siRNAs against the three genes above is shown in Table 1.

**Table 1.** Clinical studies exploring genes as potential CRPC targets

Gene	Main Results	siRNA targeting gene sequence
FOLH1	Gene ID: 2346, folate hydrolase 1, also known as PSMA Effective diagnostic and prognostic indicator of PCa Increased in PCa vs. normal control and correlated with the Gleason score in TCGA Tissue-specificity: specific high expression in PCa across TCGA tumors	GAAGCAGTTTGAAGAATTA
NKX3-1	Gene ID: 4824, NK3 homeobox 1 Role in CRPC: transcriptional activity of AR aberrant expression is associated with prostate tumor progression Increased in PCa vs. normal control in TCGA Tissue-specificity: specific high expression in PCa across TCGA tumors	ACTTGGAGAAGCACTCCTCTT
DHRS7	Gene ID: 51635, dehydrogenase/reductase 7 Role in CRPC: steroid synthesis and metabolism Increased in PCa vs. normal control in TCGA Tissue-specificity: specific high expression in PCa across TCGA tumors	GAGCTTAACTACTTAGGGA

#### Down-regulation effect of target genes and cell apoptosis

**Figure 6.**
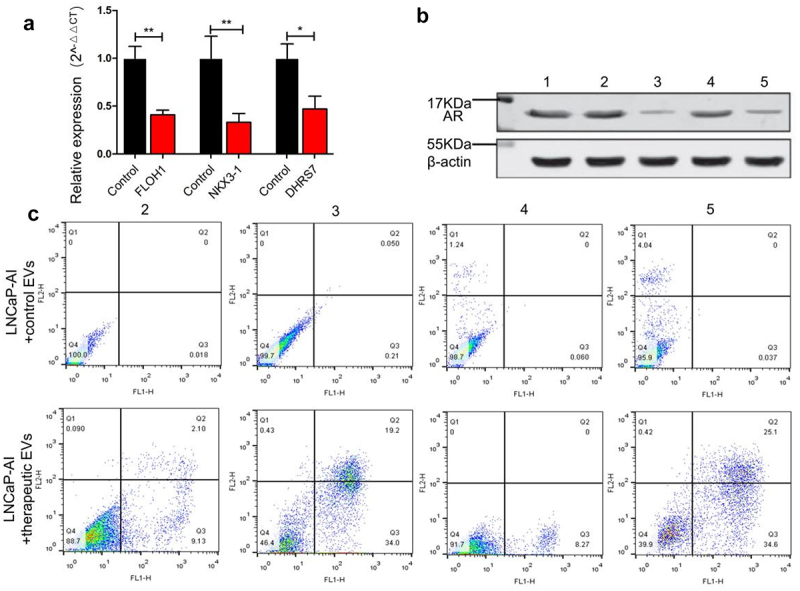
The therapeutic effect of engineered EVs on LNCaP-AI cells. (a) Expression of siRNA-targeted genes was measured by qRT-PCR. Black and red bars represent LNCaP-AI cells treated with control EVs (loading siRNA-NC) and engineered EVs (loading therapeutic siRNA mixture against FLOH1, NKX3 and DHRS7 genes), respectively. (b) Western blot analysis of AR protein expression. LNCaP-AI cells were treated with various engineered EVs groups as below. 1: untreated cells; 2: +engineered EVs loaded siRNA against FLOH1; 3: +engineered EVs loaded siRNA against NKX3-1; 4: +engineered EVs loaded siRNA against DHRS7; 5: +engineered EVs loaded siRNAs mixture against FLOH1, NKX3, DHRS7 in equal proportions. (c) Flow cytometry analysis of LNCaP-AI apoptosis after incubation with various engineered EVs grouped as (B).

To evaluate the down-regulation effect of siRNAs delivered by engineered EVs, we constructed therapeutic EVs by mixing three different siRNAs that target FLOH1, NKX3, and DHRS7 in equal proportions. qRT-PCR analysis revealed that the mRNA level of the three genes was decreased dramatically, respectively (P < .05), in LNCaP-AI cells relative to the untreated cells (Figure 6a). The treatment effect was further verified by determining AR expression using Western blot. The AR protein level was decreased by 76.2% in the therapeutic EVs group compared with the control EVs group (Figure 6b). Inhibition of three genes is expected to induce cell apoptosis since they are essential factors promoting CRPC cell proliferation and progression. Using flow cytometry, we observed a significant increase in apoptosis by the therapeutic EVs group compared with the control group. Consistent with prediction, engineered EVs loaded with multiple-targeted siRNAs showed a better apoptosis-inducing effect (61.5%) than the single target siRNA groups (FLOH1 11.23%, NKX3 53.2%, DHRS7 8.27% respectively) (Figure 6c).

## Discussion

siRNAs have been extensively used for selective gene down-regulation in tumor treatment.^21,22^ However, safe delivery systems are needed to solve their poor cellular uptake, instability, and immunogenicity in the biological environment.^23^ EVs-based delivery is a subject of intensive research, while its therapeutic application is hampered by the lack of efficient and reproducible loading methods for RNA drugs. Currently, electroporation is the mainstay of exogenous siRNA loading strategy but with irreversible damage to the EVs membrane.^24^ To overcome these limitations above, we exploited the polycationic membrane-penetrating peptide TAT to encapsulate siRNAs into EVs. Three TAT peptides were expressed with DRBD as 3TD fusion protein in an E. coli prokaryotic expression system. DRBD binds with therapeutic siRNAs in a sequence-independent manner, which facilitates multiplex gene targeting. This is particularly important in the treatment of CRPC with a variety of crucial genes involved in the androgen resistance process.

To our knowledge, this is the first study on the application of TAT for EVs siRNA loading. It is also the first study that combined siRNAs targeting androgen resistance genes in CRPC was used to develop engineered EVs. The high uptake efficiency of engineered EVs into cells and the noticeable down-regulation effect of target genes suggested the potential of TAT-DRBD as efficient siRNAs carriers by keeping the function of the cargoes. Moreover, cell apoptosis assay confirms that multi-target interference is superior for a single target in CRPC.

The limitation of the present study was the insufficient yields and tissue-specific targeting of EVs. To obtain sufficient EVs for siRNA loading, WPMY-1 cells were repeatedly sub-cultured to obtain sufficient EVs-rich conditioned supernatant for UC-based isolation, which is laborious and time-consuming. One of the major limitations of its clinical application is how to produce EVs fast and efficiently. So far, some studies have previously developed bioinspired EVs-mimetic nanovesicles by the extrusion of cultured cells using serial diminishing pore size filters with 100-fold higher yield.^25^ Tissue-specific delivery is also an important consideration for optimization. To achieve targeting, some studies have reported to engineer cells to express tissue-specific protein fused to the exosomal membrane protein.^26,27^ Although these artificially engineered nanovesicles whether have similar characteristics to natural secreted EVs is unclear, methods to increase production and targeting are worth trying in the future study.

In conclusion, our data have provided proof of concept for the functional membrane-penetrating peptide TAT as an effective natural means for delivering RNA interference drugs into EVs. The simultaneous siRNA-mediated knockdown of FLOH1, NKX3, and DHRS7 genes shows great potential for improving CRPC treatment.
